# Vanadyl Phthalocyanine Films and Their Hybrid Structures with Pd Nanoparticles: Structure and Sensing Properties

**DOI:** 10.3390/s20071893

**Published:** 2020-03-29

**Authors:** Darya Klyamer, Aleksandr Sukhikh, Nataliya Nikolaeva, Natalya Morozova, Tamara Basova

**Affiliations:** Nikolaev Institute of Inorganic Chemistry SB RAS, Lavrentiev Pr. 3, Novosibirsk 630090, Russia; klyamer@niic.nsc.ru (D.K.); a_sukhikh@niic.nsc.ru (A.S.); nikolaeva@niic.nsc.ru (N.N.); mor@niic.nsc.ru (N.M.)

**Keywords:** vanadyl phthalocyanine, thin films, chemiresistive sensors, palladium nanoparticles, hydrogen, ammonia

## Abstract

In this work, thin films of vanadyl phthalocyanines (VOPc and VOPcF_4_) are studied as active layers for the detection of gaseous ammonia and hydrogen. The effect of F-substituents on the structural features of vanadyl phthalocyanine films and their sensor response toward ammonia (10–50 ppm) and hydrogen (100–500 ppm) is investigated by X-ray diffraction (XRD) and chemiresistive methods, respectively. It is shown that the sensor response of VOPcF_4_ films to ammonia is 2–3 times higher than that of VOPc films. By contrast, the sensor response to hydrogen is higher in the case of VOPc films. Apart from this, the hybrid structures of vanadyl phthalocyanine films with Pd nanoparticles deposited on their surface by a chemical vapor deposition method are also tested to reveal the effect of Pd nanoparticles on the sensitivity of VOPc films to hydrogen. Deposition of Pd nanoparticles on the surface of VOPc films leads to the noticeable increase of their sensitivity to hydrogen.

## 1. Introduction

Among numerous organic semiconductors, metal phthalocyanines (MPc) are of particular interest because of their high thermal and chemical stability combined with their unique electronic properties. Being stable organic semiconductors, MPcs are used in energy-conversion devices (photovoltaic and solar cells), optical devices and as active layers of chemical sensors [1,2,3,4]. Films of MPcs are widely used as active layers of chemiresistive sensors [5,6]. Among them, vanadium phthalocyanine derivatives which have different single crystal structure and phase transition in thin films due to their non-planar structure, were shown [7,8] to exhibit chemiresistive sensor response to NO_2_ and humidity.

The phase composition, morphology and orientation of MPc molecules in thin films are of particular importance for their use in sensing devices because they determine both optical and electrical properties of the films [9,10]. It is well known that the choice of deposition conditions can significantly affect the growth process and the molecular organization of thin organic films [11,12,13]. So far, significant efforts have been made to develop growth methods and, ultimately, to control the structure (molecular orientation, polymorphism and morphology) of vanadyl phthalocyanine (VOPc) films. Two main polymorphs with monoclinic (phase-I) and triclinic structures (phase-II) were resolved by Griffiths et al. for VOPc [14,15]. It was shown that the phase composition of VOPc films depended both on substrate temperature [16,17] and on evaporation rate [18]. It was found that VOPc films deposited at room substrate temperature had a monoclinic structure, while they transformed to triclinic phase during post deposition annealing above 100 °C or formed it at the substrate temperature about 300 °C during evaporation [16,17,19]. 

The sensing properties of metal phthalocyanines can also be strongly affected by substituents in benzene rings, e.g., the introduction of electronegative fluorine atoms as well as fluroalkyl and fluoroaryl groups is a powerful tool for tuning MPc properties, especially electrical and sensor characteristics of their films [20,21]. It was shown in previous works [22,23,24] that the introduction of electron-withdrawing fluorine substituents led to an increase of the oxidation potential of the phthalocyanine molecule and, as a consequence, to an increase of the sensitivity of fluorosubstituted metal phthalocyanines to reducing gases like NH_3_ and H_2_. 

The synthesis and electrochemical properties of VOPcF_16_ were described by Handa et al. [25]. Schlettwein et al. investigated the epitaxial growth of VOPcF_16_ films on NaCl, KCl, and KBr substrates [26]. It was shown in our previous works [27] that VOPcF_16_ films underwent phase transition upon annealing in the temperature range of 20–220 °C. It is necessary to mention that at that time the structure of VOPcF_16_ single crystals was not refined and the conclusions about the film orientation were based mainly on the data of polarized Raman spectroscopy. To the best of our knowledge, tetrafluorosubstituted vanadyl phthalocyanine (VOPcF_4_) and its thin films have never been studied in the literature. 

Apart from single-component phthalocyanine films, bilayered structures or MPc films decorated with metal nanoparticles are used for sensing applications. Application of metal nanoparticles with catalytic properties (e.g., Pt, Pd, Au) leads to an increase in the sensitivity and selectivity of sensing layers due to the so-called spillover effect, i.e., activation of gas molecules on a metal catalytic particle and their diffusion into a gas-sensitive semiconductor layer [28]. This effect was described for metal oxide layers with noble metals [29]. For instance, the sensitivity of Pd (0.2%) doped mesoporous SnO_2_ to hydrogen was almost 10 times higher than that of pure SnO_2_ (H_2_ concentration was 1000 ppm at 250 °C) [30]. Thin SnO_2_ films covered with palladium islands (H_2_ concentration was 200–250 ppm at 400 °C, response time was 14 s) were obtained by means of magnetron sputtering by Toan et al. [31]. Fardindoost et al. [32] studied the sensor properties of thin WO_3_ films with Pd nanoparticles obtained by the sol-gel method; an increase of the amount of Pd nanoparticles led to the improvement of the films’ sensitivity in the temperature range from 30 to 350 °C due to a decrease in the size of WO_3_ crystal grains and electronic sensitization effect at the Pd/WO_3_ interface.

At the same time, the works devoted to the application of systems on the basis of MPcs and Pd nanoparticles are not numerous. Jakubik et al. [33,34,35] used surface acoustic wave (SAW) sensors based on bilayer structures consisted of MPc films and Pd layers for hydrogen detection. The Pd layers were deposited on the surface of MPc films by a physical vapour deposition (PVD) technique. In our previous paper [36], bilayer structures PdPc/Pd with the Pd layer deposited by a chemical vapor deposition (CVD) technique were shown to be good candidates for the selective detection of hydrogen (1–10 v.%) in the presence of NH_3_, CO_2_, NO_2_ and chlorinated alkane vapours.

In this work, thin films of VOPc and VOPcF_4_ are studied as active layers for the detection of gaseous ammonia and hydrogen. The effect of F-substituents on the structural features of vanadyl phthalocyanine films and their chemiresistive sensor response toward ammonia (10–50 ppm) and hydrogen (100−500 ppm) is investigated. Apart from this, hybrid structures of vanadyl phthalocyanine films with Pd nanoparticles deposited onto their surface by a pulse-CVD method are also tested and the effect of Pd nanoparticles on their sensitivity to hydrogen is studied.

## 2. Materials and Methods

### 2.1. Phthalocyanine Films Preparation 

VOPc was synthesized according to the procedure described elsewhere [37] from phthalonitrile and VCl_3_. VOPcF_4_ was synthesized according to the same technique using 4-fluorophthalonitrile (99%, Aldrich, St. Louis, MO, USA) as an initial organic reagent. VOPc and VOPcF_4_ films were prepared by an organic molecular beam deposition technique at the substrate temperature of 80 °C and residual pressure of 2·10^−5^ Torr. The deposition rate was 0.06 nm⋅s^−1^. The films thickness was determined by the method of spectral ellipsometry to be in the range of 100−120 nm. Glass slides with deposited Pt interdigitated electrodes (IDE) were used as substrates for the investigation of sensor properties.

### 2.2. Deposition of Pd Nanoparticles

**Figure 1:**
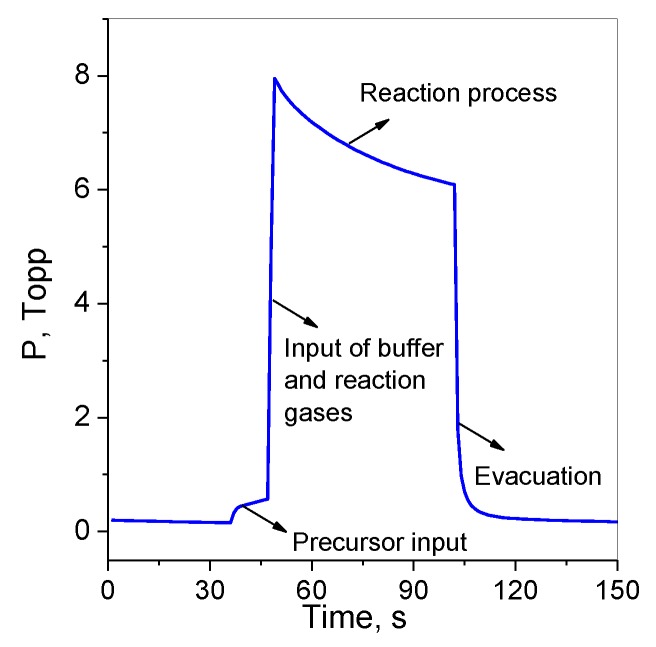
Change of the pressure during the deposition cycle.

Pd nanoparticles were deposited on the substrates with Pt interdigitated electrodes, preliminarily coated with VOPc or VOPcF_4_ films, by a CVD method with the system of pulse gas dosing of the precursor vapor and reactant gas. Palladium hexafluoroacetylacetonate Pd(hfac)_2_ was used as a volatile precursor. The experimental parameters of deposition were the following: the vaporization temperature (T_vap_) was 70 °C; the substrate temperature (T_s_) was 250 °C; the ratio of buffer-gas/reactant gas (Ar/H_2_) was 4:1,6; the pulse cycles number was varied from 20 to 40; the total reactor pressure was ~8 Torr. The deposition cycle involves the following steps: evacuation of the reactor, input of the precursor, input of the buffer and reaction gases, decomposition reaction and evacuation of the reactor. The change of the pressure during the deposition cycle is shown in Figure 1.

### 2.3. Characterization of Thin Films

X-ray diffraction (XRD) patterns of polycrystalline powder were obtained using a Shimadzu XRD-7000 powder diffractometer (Cu-anode sealed tube, Bragg-Brentano geometry, θ-θ goniometer, scintillation counter) with 0.03° 2θ scan step and 30 s accumulation time for each step. Thin films were studied using the combination of instruments, namely XRD-7000 for standard powder patterns and Bruker DUO single-crystal diffractometer (APEX II charge-coupled device (CCD) detector, CuKα, Incoatec IμCu microfocus source) for 2D GIXD (two-dimensional grazing incidence X-ray diffraction) patterns using a special sample adaptor. The primary beam angle of incidence was in the range from 0.2 to 0.4°. The distance from a sample to a CCD detector was 80 mm. 2D GIXD method has already been described by Sukhikh et al. [38,39].

The samples microstructure was investigated using a scanning electron microscope (SEM) JEOL–JSM 6700 F. Photoelectron spectra of Pd nanoparticles were recorded using a SPECS spectrometer with a Phoibos - 150 MCD-9 hemispherical energy analyzer and X-ray monochromator Focus 500 (Al Kα; hν = 1486.7 eV). The operational pressure was ~2·× 10^−8^ Torr. Pass energy of electron energy analyzer was 50 eV. Ion sputtering was done in situ for 30 min using 2.5 keV Ar+ ions beam.

### 2.4. Study of Sensor Properties

The sensor response toward hydrogen (10−500 ppm) and ammonia (10−50 ppm) diluted in air was studied by a chemiresistive method. Phthalocyanines and their bilayered structures with Pd nanoparticles were deposited on platinum interdigitated electrodes (DropSens, Oviedo, Spain) to investigate their resistance changes upon interaction with the gaseous analytes. The dimension of gaps was 10 μm; the number of digits was 125 × 2 with a digit length equal to 6760 μm; cell constant was 0.0118 cm^−1^. Pure commercial H_2_ and NH_3_ gases were used as analyte sources. Gases-analytes were injected into the flow cell at a constant flow rate of 300 ml/min; the exposure time was 15 s. After each input of a gas-analyte of a certain concentration the cell was purged with air. The electrical resistance of films was measured using a Keithley 236 electrometer by applying a constant dc voltage of 10 V. 

## 3. Results and Discussion

### 3.1. Structure of Vanadyl Phthalocyanines (VOPc and VOPcF_4_) Thin Films

**Figure 2:**
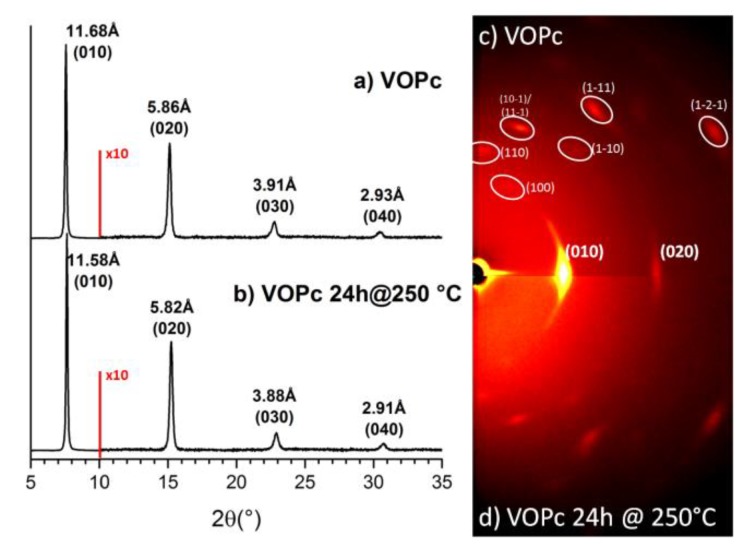
X-ray diffraction (XRD) patterns of vanadyl phthalocyanine (VOPc) films: as-deposited (**a**), after annealing for 24 h at 250 °C (**b**). The range from 10° to 35° 2θ is shown with 10-fold magnification. Two-dimensional grazing incidence X-ray diffraction (2D GIXD) patterns of the films before (**c**) and after annealing (**d**).

Structure of VOPc and VOPcF_4_ films prepared by organic molecular beam deposition was studied by XRD. An XRD pattern of VOPc film is shown in Figure 2. The inset shows the 2D GIXD pattern of the same film. The XRD pattern contains one very strong diffraction peak at d_0_ = 11.68Å and three weak peaks with interplanar distances equal to d_0_/2, d_0_/3 and d_0_/4. It indicates that VOPc crystallites have strong preferred orientation relative to the substrate surface.

The same conclusion can be made according to the 2D GIXD image. This image has several bright localized diffraction spots instead of uniform diffraction arcs with the positions corresponding to the triclinic-II polymorph of VOPc [14]. In contrast to our data Pan et al. [16] observed the growth of VOPc films with a monoclinic structure (phase-I) when deposited on a substrate at room temperature. At the same time, Minami and Asai [18] found that both structures could be formed at room substrate temperature, depending on the evaporation rate, i.e., triclinic—at 0.05 nm/s and monoclinic—at 9 nm/s. The rate of VOPc films growth was 0.06 nm/s in our case. 

Vanadyl phthalocyanine films were then used for the preparation of bilayer structures in which Pd nanoparticles were deposited on their surface by a pulse-CVD method. During pulse-CVD of Pd nanoparticles vanadyl phthalocyanine films were heated to about 200 °C. It is well known [40,41] that heating of phthalocyanine films in the temperature range from 150 to 250 °C led to phase transitions. For this reason, the effect of heating on the structural features was also studied.

Annealing of VOPc films at 250 °C results in a slight change in interplanar distances (max. 0.1Å), an increase in peak intensity, and a decrease in FWHM (full width at half maximum) (e.g., for (010) peak 5.5·× 10^5^ counts and 0.136° for the as-deposited film vs. 6.1·× 10^5^ and 0.125° for the annealed ones). These changes may be attributed to the improved crystallinity of the VOPc film after heating.

**Figure 3:**
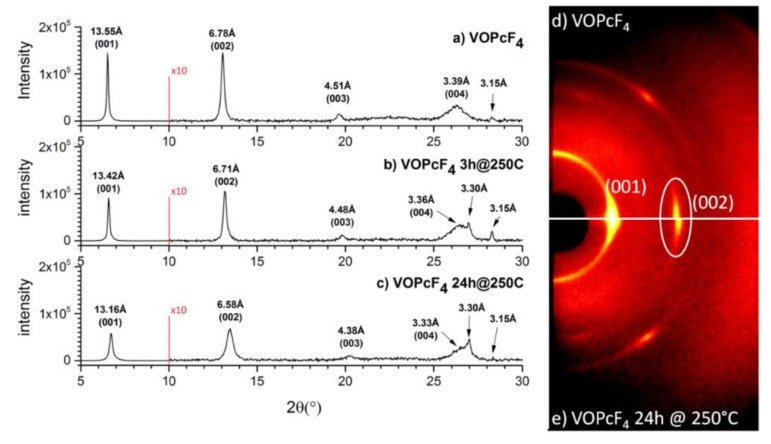
XRD patterns of a VOPcF_4_ film: as-deposited (**a**), after annealing for 3 h at 250 °C (**b**) and for 24 h at 250 °C (**c**). The range from 10° to 30° 2θ is shown with 10-fold magnification. 2D GIXD patterns before (**d**) and after annealing (**e**).

XRD and 2D GIXD patterns for VOPcF_4_ film are shown in Figure 3. Similarly to VOPc, the XRD pattern of VOPcF_4_ film has few diffraction peaks with multiple interplanar distances, indicating its strong preferred orientation. VOPcF_4_ was shown in our previous work [42] to crystallize during sublimation in vacuum as a triclinic polymorph. The structure of only one VOPcF_4_ polymorph was determined. A comparison of the peak positions on XRD and 2D GIXD patterns of the as-deposited VOPcF_4_ film with the calculated VOPcF_4_ powder pattern [42] shows that the as-deposited film has phase composition different from the powder. First, on the calculated VOPcF_4_ powder pattern the (002) peak has very low intensity, while its intensity is noticeably higher on the film diffraction pattern. Second, the 2D GIXD pattern for the triclinic space group should have more reflections than we can see in Figure 3. Therefore, the as-deposited VOPcF_4_ film appears to have unknown crystal structure different from the triclinic polymorph. 

In contrast to VOPc, the XRD pattern of VOPcF_4_ changes noticeably after the film annealing at 250 °C. The XRD patterns of the as-deposited VOPcF_4_ film and the same films after annealing at 250 °C for 3 and 24 h are shown in Figure 3b,c. The position of all observed peaks (except the weak peak at 3.15 Å) shifts to the higher 2Θ angles, indicating a more noticeable decrease of VOPcF_4_ unit cell parameters than in the case of VOPc (cf. maximum shift 0.1 Å for VOPc and 0.39 Å for VOPcF_4_). Moreover, their intensities significantly decrease and FWHM increase (e.g., for the (001) peak 1.5·10^5^ counts and 0.107° for the as-deposited film vs. 5.7·10^4^ and 0.192° for the annealed film). These changes can be attributed to the alterations in the VOPcF_4_ crystal lattice (VOPcF_4_ molecules shift relative to each other and/or change their orientation). Additional evidence in favor of structural changes is the appearance of the new peak at 3.3 Å on the XRD pattern after annealing, corresponding to the most intense peak (2 –2 –2) of the triclinic phase [42]. These facts testify that annealing appears to cause a transition of VOPcF_4_ from an unknown low-temperature crystal phase to triclinic one, and the annealed VOPcF_4_ film consists of the unknown crystal phase with the admixture of triclinic phase. A phase transition upon annealing was also observed in VOPcF_16_ thin films [27].

### 3.2. Sensor Response of VOPc and VOPcF_4_ Films to Ammonia and Hydrogen

**Figure 4:**
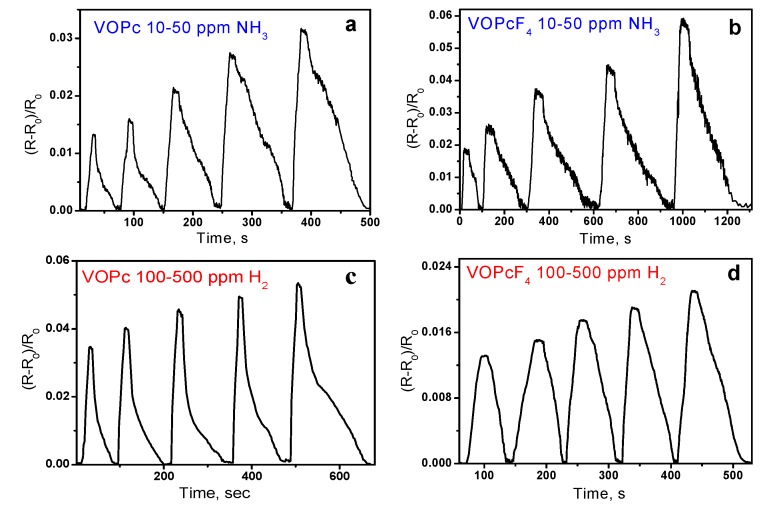
Sensor response of VOPc and VOPcF_4_ films to ammonia (**a**,**b**) and hydrogen (**c**,**d**).

**Figure 5:**
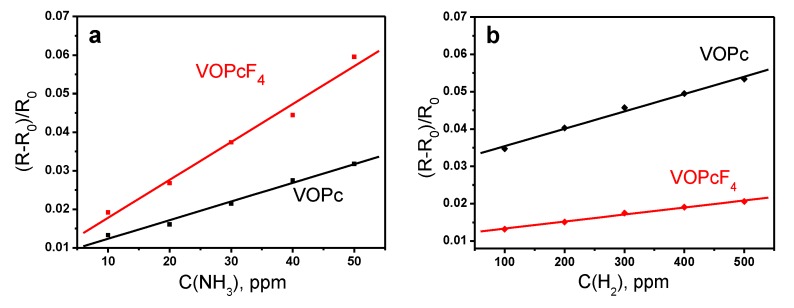
Dependence of the sensor response on ammonia (**a**) and hydrogen (**b**) concentrations for VOPc, VOPcF_4_ films.

The sensor properties of VOPc and VOPcF_4_ thin films toward ammonia and hydrogen were investigated by a chemiresistive method. Phthalocyanine films deposited on substrates with interdigital electrodes were placed in the flow cell in which ammonia (10–50 ppm) or hydrogen (10–500 ppm) were injected and the change of films’ resistance was continuously monitored. The typical sensor responses as the dependence of (R-R_o_)/R_o_ (where R is the steady resistance of the film at a certain analyte concentration and R_o_ is the baseline resistance of the film) on time are shown in Figure 4. The introduction of both ammonia and hydrogen to the gas cell led to an increase of the resistance of VOPc and VOPcF_4_ films. The sensing mechanism of the semiconducting sensors has already been studied in the literature [43]. All investigated films demonstrated completely reversible sensor response to ammonia and hydrogen at room temperature. The dependence of sensor response on analyte concentration is shown in Figure 5. 

The sensor response of VOPcF_4_ films to ammonia is 2–3 times higher than that of VOPc films. By contrast, the sensor response to hydrogen is higher in the case of VOPc films. Similar behavior was also observed in the case of PdPc and PdPcF_16_ films and explained by different mechanisms of resistance change upon interaction of phthalocyanines with NH_3_ and H_2_ that was described with the use of DFT calculation by Parkhomenko et al. [44]. Ammonia interacts with the central metal ion inside the phthalocyanine ring and forms complexes with charge transfer from NH_3_ to phthalocyanine molecule. It was shown in previous publications [24] that the introduction of electron-withdrawing fluorine substituents led to the increase of the sensor response of MPcF_4_ derivatives in comparison with their unsubstituted analogues. Another factor influencing the sensor response is the film crystallinity. It is known that the crystal size plays an important role [45,46]. The larger crystal size results in smaller surface area and fewer adsorption sites for analytes, resulting in a smaller sensor response as compared to that of the films composed of much smaller granular crystals. The same is observed for amorphous films [45] which with the looser molecular stacking provide more adsorption sites and show the higher sensor response [46]. The lower degree of crystallinity of VOPcF_4_ films may also contribute to their higher sensor response to ammonia than in the case of VOPc ones. The change of MPc films’ resistance upon interaction with H_2_ has different mechanism from that occurring in the case of ammonia and results from a gain of surface electrons following the reaction of hydrogen with adsorbed oxygen.

The response and recovery times of VOPc and VOPcF_4_ films are presented in Table 1. The response and recovery times for VOPcF_4_ were higher than those of VOPc films for the same ammonia concentrations, while in the case of hydrogen the response time of VOPcF_4_ was two times less than for VOPc.

### 3.3. Hybrid Structures of VOPc with Pd Nanoparticles

It has already been shown [28,29] that application of hybrid materials of semiconductors with metal nanoparticles (e.g., Pt, Pd, Au) led to an increase in the sensitivity of sensing layers to hydrogen. In our previous paper [36], bilayer structures in which a Pd layer (55–160 nm in thickness) was deposited on the surface of palladium phthalocyanine film by a pulse-CVD technique were shown to be promising active layers of chemiresistive sensors for selective detection of hydrogen. However, the sensing performance was studied against high hydrogen concentrations of 1–30 v.% in air. In this work, Pd nanoparticles were deposited on the surface of VOPc films by a pulse-CVD technique and the sensor response of the prepared heterostructures to hydrogen was tested. 

**Figure 6:**
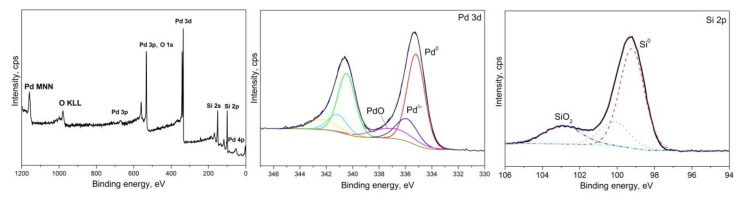
X-ray photoelectron spectroscopy (XPS) spectra of a Pd nanoparticles on a Si(100) substrate after etching with Ar+ ions.

The X-ray photoelectron spectroscopy (XPS) method was used to examine the surface state of Pd nanoparticles deposited on Si (100) at the same conditions of pulse-CVD process. Pd 3d and Si 2p XPS core-level spectra are shown in Figure 6. The Pd^0^3d5/2 level spectrum shows three Pd-related species, viz. metallic Pd^0^ (Binding Energy (BE) = 335,2 eV), Pd^2+^ corresponding to surface PdO (BE = 337,0 eV) and intermediate species of Pd^δ+^ (BE=335,9 eV) arising probably from the interaction of the surface PdO with Pd on the cluster boundary [36,47,48]. Three Si-related species at 99.2, 100.1 and 102.9 eV can be determined for Si^0^ 2p core level binding energy (Si^0^ of the substrate, partially oxidized Si^+^ and SiO_2_ (Si^4+^) of the native oxide layer, respectively) [49]. 

**Figure 7:**
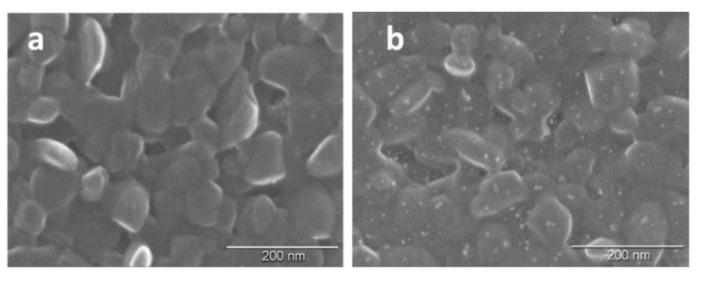
Surface morphology of a VOPc film (**a**) and VOPc film covered with Pd nanoparticles (20 cycles) (**b**).

The surface microstructure of a VOPc film covered with Pd nanoparticles (20 cycles of pulse-CVD) is shown in Figure 7 in comparison with the as-deposited VOPc film. The surface of the VOPc film consists of grains of 30–70 nm in size. Figure 7b shows that its morphology does not almost change after deposition of Pd nanoparticles. Pd nanoparticles look like light dots uniformly distributed on the surface of VOPc. The number of palladium nanoparticles increases with the increase of the number of pulse-CVD cycles.

**Figure 8:**
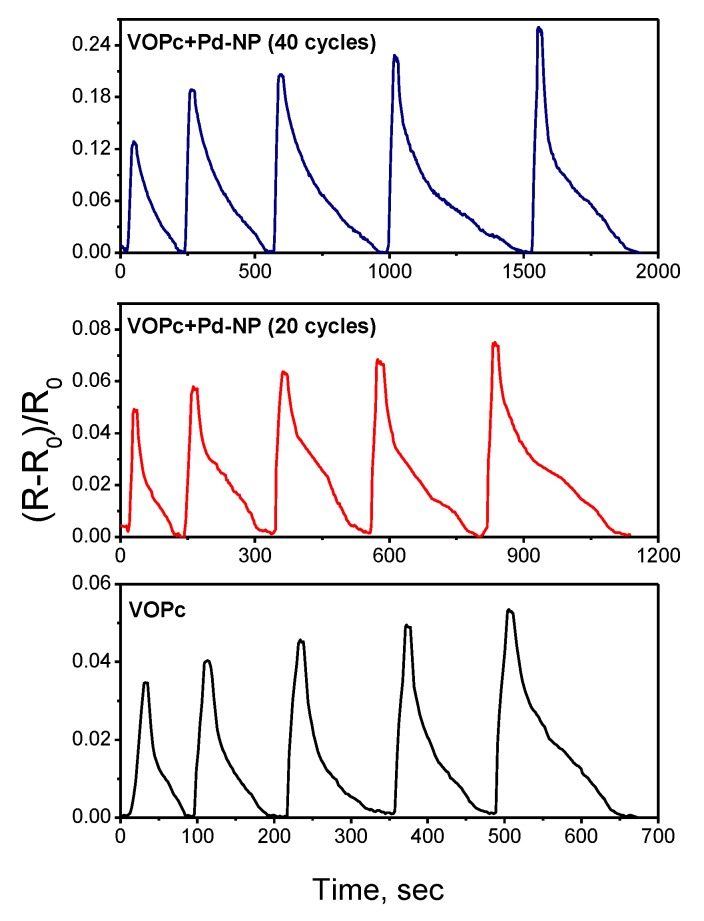
Sensor response of a VOPc film and a VOPc film covered with Pd nanoparticles during 20 and 40 cycles of pulse-chemical vapor deposition (CVD) to hydrogen (100–500 ppm), measured at 80 °C.

**Figure 9:**
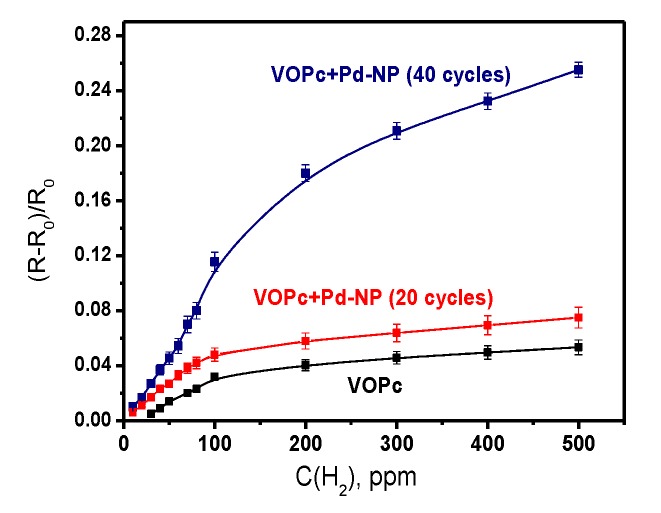
Dependence of the sensor response of a VOPc film and VOPc films covered with Pd nanoparticles during 20 and 40 cycles of pulse-CVD on hydrogen concentration.

In contrast to the VOPc films, the sensor responses of VOPc films covered with Pd nanoparticles, measured at room temperature, was not completely reversible. For this reason, all further investigations of the sensor response to hydrogen were carried out at 80 °C. The sensor responses of VOPc films and VOPc films covered with Pd nanoparticles to hydrogen (100–500 ppm), measured at 80 °C, are shown in Figure 8, whereas the dependence of these sensor responses on hydrogen concentration (10–500 ppm) is presented in Figure 9.

Both figures show that the VOPc films with Pd nanoparticles deposited during 20 cycles of pulse-CVD exhibits the 1.4-fold increase of the sensor response to hydrogen, while the sensor response of the film with more Pd nanoparticles obtained during 40 pulse-CVD cycles increases by 4–5 times. Deposition of nanoparticles on the surface of VOPc films leads to the noticeable increase of their recovery time, *viz.* the recovery time measured at 300 ppm of hydrogen increases from 18 s for VOPc to 165 s for VOPc + Pd-NP (20 cycles) and 370 s for VOPc + Pd-NP (40 cycles) heterostructures. The detection limit of hydrogen is 30 ppm in the case of the VOPc film, while it decreases to 10 ppm in the case of VOPc + Pd-NP heterostructures.

It is known that in the case of composite materials of semiconducting oxides, Pd nanoparticles are used to enhance gas sensing performance due to their improved catalytic activity, resulting from the increased surface area and “spill-over” effect [50,51]. When hydrogen is exposed to a semiconducting layer, the H_2_ molecules dissociate into more active atomic hydrogen H in the presence of catalytic Pd nanoparticles, which spills over to the surface of semiconductor film and interacts with adsorbed oxygen with the release of electrons. The variation of the surface electron depletion region in the case of a p-type semiconductor or hole accumulation region in the case of a n-type semiconductor due to the reaction between hydrogen and chemisorbed oxygen on the surface leads to the change of film resistance. Most likely a similar process proceeds in the case of MPc/Pd heterostructures. 

The operation temperature is a unique characteristic of each sensor. The increase of operation temperature to 80 °C does not influence the value of sensor response of a VOPc film without nanoparticles; only a small decrease of the recovery time becomes noticeable at 400–500 ppm of hydrogen. At the same time, the increase of operation temperature to 80 °C results in the increase of the sensor response and decrease of the recovery time of VOPc films covered with Pd nanoparticles. Temperature is an important factor that greatly influences the hydrogen sensing response based on the catalytic effect. It was shown in previous publications [52] that the higher temperature led to the higher sensing performance due to the lowering of activation energy for gas adsorption and desorption of the Pd-based sensor. Upon exposure to dry air for the recovery process dissolved hydrogen on Pd nanoparticles reacts with oxygen in air with the formation of H_2_O. At the higher working temperature, the response time is shorter due to the faster desorption of the formed water molecules at the Pd nanoparticles surface. 

**Figure 10:**
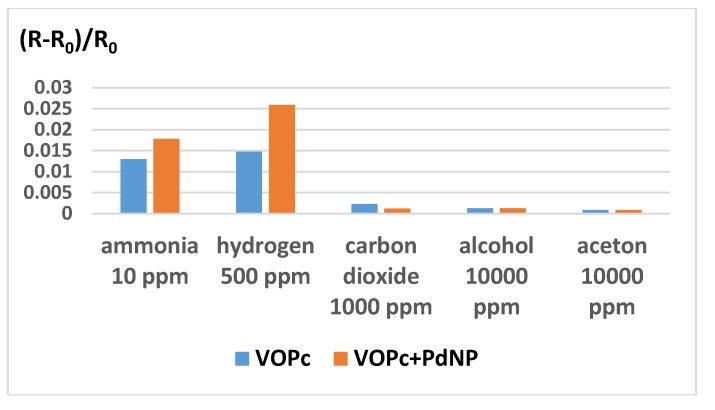
Sensitivity of both VOPc + Pd-NP structures and VOPc films to ammonia, hydrogen, carbon dioxide, alcohol and acetone.

The diagram in Figure 10 shows the sensitivity of both VOPc+Pd-NP structures and VOPc films to various gases and volatile organic vapors. Both active layers can be used for the detection of hydrogen in the presence of CO_2_, alcohols and acetone, while ammonia is an interfering gas. It is necessary to mention that the sensitivity of VOPc + Pd-NP structures to ammonia also increases in comparison with VOPc films, but this is not as prominent as in the case of hydrogen. 

Note that the sensor performance of various sensors on the basis of heterostructures of metal oxides with metal layers or nanoparticles towards hydrogen has been reported in the literature [53,54,55,56,57]. Several examples of sensor characteristics including the data obtained in this work are summarized in Table 2.

The sensing layers based on VOPc with Pd nanoparticles are quite competitive with the active layers based on the sensing materials containing noble metal nanoparticles or thin films, described in the literature. 

## 4. Conclusions

In this work, thin films of VOPc and VOPcF_4_ were studied as active layers for the detection of gaseous ammonia and hydrogen. The effect of F-substituents on the structural features of vanadyl phthalocyanine films and their chemiresistive sensor response toward ammonia (10–50 ppm) and hydrogen (100–500 ppm) was investigated. VOPc films were grown in a triclinic-II phase and their annealing at 250 °C for several hours did not lead to any visible differences in their diffraction patterns other than a slight improvement in crystallinity. At the same time, VOPcF_4_ formed films with an unknown crystal phase which transformed to a triclinic one upon heating. It was shown that the sensor response of VOPcF_4_ films to ammonia was 2–3 times higher than that of VOPc films. By contrast, the sensor response to hydrogen is higher in the case of VOPc films. 

The hybrid structures of VOPc films with Pd nanoparticles deposited on their surface were also tested to reveal the effect of Pd nanoparticles on the sensitivity to hydrogen. Pd nanoparticles were deposited by a pulse-CVD technique. It was shown that the sensor response of VOPc films with Pd nanoparticles deposited during 20 cycles of pulse-CVD exhibited a 1.4-fold increase of the sensor response to hydrogen, while the sensor response of the film with more Pd nanoparticles obtained during 40 pulse-CVD cycles increases by 4–5 times.

## Figures and Tables

**Table 1:** The response and recovery time (s) of VOPc and VOPcF_4_ films toward 30 ppm of ammonia and 300 ppm of hydrogen.

Response/Recovery Time	NH_3_ (30 ppm)	H_2_ (300 ppm)
VOPc films	15/65	18/100
VOPcF_4_ films	36/230	20/50

**Table 2:** Characteristics of various sensors for hydrogen detection.

Sensing Layer	C(H_2_) (ppm)	Response/ Recovery Time (s)	Temperature Range (°C)	Ref.
Pt-WO_3_ with an amorphous SiO_2_ layer	3–150	18/349 (150 °C, 150 ppm)	100–350	[53]
Pt-decorated SnO_2_ nanorods (Pt/Sn ratio of 3.63%)	100–1000	0.3/29.6 (room temperature (RT), 1000 ppm)	Room temperature	[54]
Pd/ZnO nanowire	100	6.4/7.4 (RT, 100 ppm)	Room temperature	[55]
Pd/Ni film	4000–20000	7/23 (75 °C, 20,000 ppm)	25–100	[56]
Pd/g-C_3_N_4_	1000–4000	88/- (30 °C, 4000 ppm)/	30–80	[57]
VOPc films with Pd nanoparticles	10–500	25/180 (100 ppm)	80	This work
